# Supplementary material for: Transcriptomic Profiling of Human Limbus-Derived Stromal/Mesenchymal Stem Cells—Novel Mechanistic Insights into the Pathways Involved in Corneal Wound Healing
Source: Int J Mol Sci. 2022 Jul 26;23(15):8226. doi: 10.3390/ijms23158226 (PMC9368612; doi:10.3390/ijms23158226)
Supplement: Supplementary file 1 [file ijms-23-08226-s001.zip › ijms-1803949-supplementary.pdf]

Supplementary Materials

Supplementary Table S1: Antibodies used in Immunostaining

Reagent or Resource	Source	Catalogue #
Rabbit polyclonal anti-PAX6	Abcam	ab5790
Mouse monoclonal anti-CD90 (Thy-1)	Santa Cruz	SC-59396
Mouse monoclonal anti-CD105 (Endoglin)	Santa Cruz	SC 376381
Mouse monoclonal anti-Vimentin	Santa Cruz	SC 6260
Mouse Monoclonal anti-ABCG2	Abcam	ab3380
Goat polyclonal anti-ABCB5	Santa Cruz	SC-104019
Rabbit polyclonal anti-p63-α	Cell Signalling	#4892
Mouse monoclonal anti-E-Cadherin	Abcam	ab1416
Mouse monoclonal anti-N-Cadherin	Santa Cruz	SC-393933

Supplementary Table S2: Primer sequences used in in qRT-PCR

Gene	Primer Sequences	
	Forward (5' to 3')	Reverse (5' to 3')
ABCB5	CCAAATCGGGGGCTGCGCATCTGTT	AGCCGCTGCTCCCCACAAATGCTA
ABCG2	GGGTTCTCTTCTTCCTGACGACC	TGGTTGTGAGATTGACCAACAGACC
CD105	CGGTGGTCAATATCCTGTCGAG	AGGAAGTGTGGGCTGAGGTAGA
CD90	CGCTCTCCTGCTAACAGTCTT	CAGGCTGAACTCGTACTGGA
COL1A1	GTCACCCACCGACCAAGAAACC	AAGTCCAGGCTGTCCAGGGATG
COL3A1	TGAAAGGACACAGAGGCTTCG	GCACCATTCTTACCAGGCTC
COL5A1	CGCTCTCCCGTCTTCCTC	CACCCTCAAACACCTCCTCA
CXCR4	GATCAGCATCGACTCCTTCA	GGCTCCAAGGAAAGCATAGA
ECAD	ATTTTTCCCTCGACACCCGAT	TCCCAGGCGTAGACCAAGA
GAPDH	ACCACAGTCCATGCCATCAC	TCCACCACCCTGTTGCTGTA
HIF1A	CAAGAACCTACTGCTAATGC	TTATGTATGTGGGTAGGAGATG
IL10	GCCTAACATGCTTCGAGATC	TGATGTCTGGGTCTTGGTTC
IL13	GAGTGTGTTTGTCACCGTTG	TACTCGTTGGTCGAGAGCTG
IL6	ATGAACTCCTTCTCCACAAGCGC	GAAGAGCCCTCAGGCTGGACTG
KERA	ATCTGCAGCACCTTCACCTT	CATTGGAATTGGTGGTTTGA
LUM	CCACCACACCTGACAGAGT	CAAGTTGATTGACCTCCAGG
MMP1	AGCTAGCTCAGGATGACATTGATG	GCCGATGGGCTGGACAG
MMP3	TTCCGCCTGTCTCAGATGATAT	AAAGGACAAAGCAGGATCACAGTT
MMP9	CACTGTCCACCCCTCAGAGC	GCCACTTGTCGGCGATAAGG
NCAD	AGCCAACCTTAACTGAGGAGT	GGCAAGTTGATTGGAGGGATG
TP63	GAGGTTGGGCTGTTCATCAT	AGGAGATGAGAAGGGGAGGA
PAX6	ATAACCTGCCTATGCAACCC	GGAACTTGAACTGGAACTGAC
ACTA2/SMA	CTCCCTGGAGAAGAGAAGAA	TTTGGTGCTGATCTGTCCCT
STAT3	ACCCAACAGCCGCCGTAG	CAGACTGGTTGTTTCCATTCAGAT
TGFB1	GAGGTGACCTGGCCACCATTCAT	TCCGCAAGGACCTCGGCTGG
TIMP1	TTGTGGGACCTGTGGAAGTA	CTGTTGTTGCTGTGGCTGAT
TIMP2	AAGCGGTCAGTGAGAAGGAGTGG	CCTTGGAGGCTTTTTTGCAGTTG

VEGF	CTACCTCCACCATGCCAAGT	GCAGTAGCTGCGCTGATAGA
VIM	GGGACCTCTACGAGGAGGAG	CGCATTGTCAACATCCTGTC
β-Actin	TCTACAATGAGCTGCGTGTG	GGTGAGGATCTTCATGAGGT

Supplementary Table S3: Software and Programs used in data analysis

Software / program	Reference Link
bcl2fastq	https://support.illumina.com/downloads/bcl2fastq_conversion_software_184.html
TopHat	http://ccb.jhu.edu/software/tophat/manual.shtml
Cufflinks	http://cole-trapnell-lab.github.io/cufflinks/cufflinks/index.html
Cuffmerge	http://cole-trapnell-lab.github.io/cufflinks/cuffmerge/
Cuffdiff	http://cole-trapnell-lab.github.io/cufflinks/cuffdiff/index.html#cuffdiff-options
EbSeq	https://bioconductor.org/packages/release/bioc/html/EBSeq.html
Enrichr	https://maayanlab.cloud/Enrichr/
AmiGO	http://amigo.geneontology.org/amigo
Genemania	http://genemania.org/
STRING analysis	https://string-db.org/

Supplementary Table S4: GO Pathway level gene expression changes with respect to whole transcriptome

Biological Process or Pathway	Number of DEGs
Apoptosis	126
Cell motility	1757
Dedifferentiation	13
Endocytosis	688
EMT	158
Epithelial motility	371
Epithelial stem cells	15
ECM	748
Inflammatory response	734
Mitochondrial biogenesis	587
Mitochondrion transport	325
Respiration	193
Stem cell maintenance	160
Stromal Cells	29
Tissue Remodelling	171
Wound Healing	559

Supplementary Table S5: DEGs (differentially expressed genes) of the wound healing pathway. DEGs of the wound healing pathway expressed by limbal stromal cells: table representing the log 2-fold change (relative to sclera) of each gene related to wound healing pathway in the corneal and limbal tissues, limbal stem cells (LMSC-P0 and LMSC-P3), and embryonic stem cell, obtained from the RNA-Seq analysis. The DEGs exclusively up-regulated in hLMSC-P3 are marked in red font.

Gene	Cornea	Limbus	LMSC-P0	LMSC-P3	ESC
NINJ2	0	0	-5.23127	0	-3.35492
PDGFB	-2.51477	0	0	-5.35556	0
HRAS	1.832786	3.367234	3.562963	0	0
LOX	0	0	-2.78266	0	0
PIK3R5	-2.26322	0	-6.62066	0	0
TFPI	0	0	0	0	-5.47078
TGFB3	-2.5733	0	-4.44443	-3.74229	-8.29898
ITGA2	0	3.183476	4.169108	0	0
MIA3	0	-2.64755	0	0	0
CASP3	0	0	3.300929	0	0
CAPN3	0	0	0	0	-3.06752
COL5A1	0.66819	-0.47126	-0.74931	3.536237	-2.50102
DCN	2.579354	0	-7.36149	0	-12.4682
VANGL2	0	0	0	-3.5835	3.47421
DGKG	-3.16601	0	0	0	-3.7629
FAM46A	0	0	-3.29385	0	-6.89736
TYRO3	0	0	0	2.546345	6.490965
PIK3R1	0	0	-2.60888	0	-4.85583
EPB41L4B	0	0	3.103199	0	0
PLAU	0	5.192434	6.294242	0	0
F10	2.745544	0	-2.8447	0	0
AJUBA	0	0	3.081285	0	0
NFE2	0	0	2.617683	0	0
CORO1B	0	0	0	0	4.127566
FAP	1.807383	0	0	5.560921	0
COL1A1	0	0	0	3.86146	-3.83313
THBS1	0	0	0	0	-7.81011
PDGFC	0	0	0	0	-3.5404
LYN	-3.50507	0	0	-2.55131	0
WAS	0	0	0	-3.74028	-2.82917
SYT7	0	0	0	5.266603	4.389419
CDH3	0	4.704429	6.615209	0	6.076329
EGFR	0	0	2.597162	0	-3.92748
PROS1	0	-2.92341	-3.12484	0	-3.37538
THBD	-2.6107	0	0	-4.26042	-8.52853
GPX1	0	2.668066	2.418445	0	3.96835

PAK1	0	0	0	0	3.61732
GPR4	0	0	-5.4891	-5.83821	-4.39659
AQP1	0	0	-4.64258	-5.70161	-10.3474
TPM1	-2.39017	0	0	0	0
ADAMTS13	2.111869	0	0	0	0
LCP2	-5.37817	0	0	0	-6.68516
SYT11	0	0	0	0	-3.90405
IL24	0	0	4.086686	0	0
DDR1	0	3.660268	3.260083	0	4.044011
GAP43	0	-2.2722	-3.34899	0	0
GNG2	0	0	-2.65049	0	0
SGCA	-4.56317	0	-4.57815	-3.07241	-5.51146
ANXA2	0	0	3.616335	0	0
NOG	0	0	0	-2.37342	0
HMGCR	0	0	2.836812	0	0
MMRN1	-7.33323	-2.37607	0	0	-9.85315
AK3	-1.9465	-2.55872	0	0	-3.53179
DOCK11	0	-2.57736	-4.19032	0	0
SAA1	0	0	-2.72692	-10.0008	-10.245
F13A1	0	0	0	-9.4405	-9.68481
F8	-2.20121	0	-4.56445	-2.83822	-5.22566
SLC11A1	0	0	-4.11502	-4.10411	-3.68568
XBP1	-2.20719	0	0	0	0
DYSF	-5.89888	0	0	-4.66874	0
DGKI	-3.41685	-3.66	-3.63496	-3.09929	-4.85191
F5	-2.04803	0	-2.18961	-2.67939	-4.18353
SERPINE1	-2.11516	0	0	0	0
FGF2	0	-2.42558	0	0	0
FZD6	-2.15041	0	0	0	0
GNA14	0	0	-3.10163	0	-4.25479
FIBP	0	0	0	2.448933	3.660078
DMTN	-2.84999	0	0	0	-3.44993
P2RY1	-3.49211	0	0	0	0
TEC	1.806587	0	0	0	0
NFE2L2	0	-2.26678	0	0	-4.73648
PRKCQ	0	0	2.426271	-3.73934	0
PIK3CA	0	-2.55483	0	0	0
PRKCB	-3.48319	0	-5.36979	0	0
DSP	0	0	3.241288	0	0
RHOB	0	0	-4.07348	-3.18475	0
IRF1	0	0	0	0	-3.91348
ENPP4	-2.0331	-2.23882	0	-4.27637	0

GNA15	0	3.650278	3.137443	-5.16112	-4.06214
ITPR1	-2.04391	0	-2.51376	-2.63272	-3.88052
VAV3	0	0	0	-4.85475	0
IL6	-7.29547	0	-4.16075	-3.2859	-9.92415
NOS3	0	0	-5.17644	-5.52846	0
VAV1	-3.2494	0	0	0	0
FERMT3	-1.71929	0	-4.61095	-3.97168	-3.05553
MAFF	0	0	0	-2.81005	-3.64575
NDNF	2.46284	0	-4.15801	-5.42261	-6.87786
SERPINA10	0	3.828114	0	0	0
HPSE	2.914521	0	0	0	0
P2RX2	0	0	0	0	3.160633
RHOG	0	2.118921	0	0	0
PAPSS2	-3.57971	-4.18829	-4.39497	0	-5.1945
ITPR3	0	2.769782	0	0	0
ITPR2	0	-2.44213	0	0	0
SPRR3	-4.81098	-2.76053	-2.46869	-6.91649	0
EDN1	-2.72981	0	0	-5.78981	-4.30824
DOCK8	-3.12418	0	0	-5.61113	-3.95624
PLAT	0	0	0	0	-3.07459
PHF21A	0	-2.47994	-2.86772	0	0
FZD7	1.895351	0	0	3.081614	3.632971
OPRM1	2.378145	0	0	0	0
ANXA1	0	0	0	0	-6.9553
CEACAM1	-2.9542	0	0	-5.49174	-3.96065
ADRA2A	-6.03673	0	-7.79257	-6.02108	0
SDC1	0	3.01652	2.998155	0	0
ADRA2C	0	3.444676	0	0	5.111435
HMG20B	0	0	0	0	3.590148
TLR4	0	0	-3.01585	0	-6.57663
PLEK	-3.47793	0	-7.01405	-6.07916	0
CD177	0	0	0	0	6.681476
MYOF	0	0	0	0	-5.36592
CPQ	0	0	-4.00862	0	-3.75957
CD36	-2.03016	-2.17335	-4.79185	-5.27811	-4.35859
ZFPM2	-4.14004	0	-2.65167	0	0
TRPC6	0	0	-2.74029	0	0
GATA6	-3.82622	0	-2.58458	0	-4.77892
LGR6	2.320525	4.748744	0	0	0
ERBB3	0	2.312442	2.282257	-2.50787	0
FGFR2	0	0	0	-4.9523	0
WNT7A	-1.5838	3.425595	8.163797	-0.59201	0

MSX2	0	3.005035	0	0	0
EREG	-2.53678	0	0	-6.26118	-6.50813
MERTK	0	0	-3.47363	-6.44044	0
DOCK9	0	0	0	0	-3.71523
ERBB2	0	0	0	0	3.528435
CD44	0	0	0	0	-5.30615
HBEGF	-2.08517	0	0	-6.05469	-5.28519
F2R	0	0	-3.16536	0	-4.07475
F3	0	0	4.887113	0	0
CFLAR	0	0	0	0	-4.68211
ACVRL1	0	0	-3.94991	0	-5.0106
SERPING1	0	0	-6.41767	-3.85804	-4.75367
SERPINA5	0	0	-7.69828	-5.30207	-6.32861
PRKCG	-2.0461	-2.86124	0	-4.11737	0
SERPINB2	0	3.121542	6.102776	-5.9998	-6.65101
S100A8	-3.83339	0	0	-6.57866	0
KLKB1	0	0	0	0	3.154892
FCER1G	-4.54678	0	-6.79315	-6.65235	-5.2082
APOD	0	0	-11.3007	-8.58788	-13.4434
IGF1	-3.97979	-3.9226	-6.4183	-3.7711	-6.22037
PROCR	-2.27369	0	0	0	0
B4GALT1	0	0	0	0	-3.77272
INSL3	0	0	0	0	3.769195
SELP	0	0	-6.28883	0	0
ITGA9	-2.10217	0	-4.442	-4.79384	0
IL1A	0	0	7.202457	0	0
F2RL1	0	2.534567	0	0	0
FBLN1	-4.55666	0	-4.48429	0	0
MYLK	-4.91066	-2.86047	0	0	-3.68882
ELK3	0	0	0	0	-3.81392
VWF	-7.16082	0	-6.67973	-6.19125	-6.80509
CCNB1	0	0	6.304379	4.316678	6.203304
CSRP1	0	0	0	0	-7.19212
MMP12	-3.05423	0	-3.12046	-6.37566	0
FOXC2	-3.04762	0	-4.57513	-2.38727	0
A2M	-5.86123	0	-5.36331	-5.30088	-9.57282
JMJD1C	-2.7686	-2.94745	-2.71119	-3.11511	0
P2RX7	0	0	-2.45544	0	-3.42762
KLK6	0	0	10.39122	0	0
ADRA2B	-4.41813	0	-5.3317	0	0
ALOX12	0	0	0	0	2.984507
TNFAIP3	0	0	0	-4.74787	-7.59542

NOV	-2.74528	0	-5.17591	0	-4.25806
PIK3CG	-2.52735	0	-5.84927	0	0
TIMP1	-0.19688	-0.43101	-1.27099	4.03621	-2.39514
SERPINA1	-3.51665	0	0	-4.14847	-5.59633
MCAM	-4.84744	0	0	-3.98641	0
GSN	0	0	0	0	-5.99734
SOX2	-2.3027	0	0	0	6.134517
CYR61	0	0	-3.19586	-3.43559	-5.71079
CD109	0	0	0	0	-3.8075
PTPN12	0	-2.62354	0	0	0
SDC4	0	2.567824	0	0	0
LCK	0	0	0	0	5.504203
HPS6	0	0	2.800695	0	4.250846
UBASH3B	0	0	2.841311	0	0
RHOC	0	0	2.463326	0	0
PDPN	0	0	-2.55325	-3.42492	0
PDGFRA	0	0	-3.93987	0	-9.7373
ETS1	-3.13542	0	0	0	-4.04023
STXBP3	0	-2.15029	0	0	0
HIST1H3A	0	0	0	0	4.88989
ARHGEF19	0	0	0	0	3.281424
WNT5A	2.665635	0	0	0	-4.82207
FZD9	0	3.19743	0	0	6.015469
CYP4F11	1.821227	0	0	0	0
CX3CL1	-3.42449	0	0	-5.5505	-6.01168
PKM	0	2.483063	3.375072	3.080076	0
WNT4	0	2.372188	0	-2.99886	0
WNT3A	0	3.795823	0	0	0
DGKZ	0	0	0	0	3.804195
TNC	-5.59184	0	0	0	-7.55184
BCL9	-2.2746	0	0	0	0
TFPI2	-3.25866	0	4.549003	0	0
SCARB1	0	0	2.829801	0	3.171203
SYK	0	0	0	-5.00116	0
PRKAR2B	-2.93367	-3.22655	-4.19524	0	0
PRKAR1B	0	0	2.34458	0	4.198816
IFRD1	0	0	-2.77157	-2.55441	0
RAB27A	0	-2.75757	0	0	-3.77985
C1QTNF1	-2.90975	0	0	-2.63697	-6.50289
P2RX6	2.19859	0	0	0	0
FGF10	1.955867	0	-4.22119	0	0
PRKCH	-3.31612	0	0	-5.55223	0

ADAM15	0	0	2.703885	0	0
EHD3	2.968711	0	2.312954	3.008271	0
HBB	-5.7002	-2.23384	-4.12788	-3.61858	-3.48822
GRHL3	0	3.762522	3.335591	-4.47326	0
MAP3K5	-4.25796	-2.51692	0	0	-4.59836
METAP1	0	0	2.61445	0	0
EZH2	-1.84181	0	0	0	0
ITGB3	-3.43008	0	0	0	-6.4376
RAC2	-3.57568	0	2.473802	0	0
CD59	0	0	0	0	-3.38667
ENO3	0	0	0	0	4.09129
ANXA8	0	3.409807	3.695579	0	0
CD34	2.787035	0	-6.91657	-5.51743	-7.8455
EPPK1	0	3.936338	2.819558	-4.27339	0
CCL20	-2.88764	0	0	0	0
ITGA2B	0	0	0	0	4.201197
DGKH	0	0	0	0	-3.02228
AGER	0	0	-2.35574	0	0
SCUBE1	-5.84013	0	0	0	-4.41072
F2RL2	-2.9459	0	-4.57929	0	-7.06057
PRKG1	0	-2.20661	-3.53969	0	0
P2RX1	0	0	-3.21562	-3.07187	0
NRG1	0	3.455654	5.69539	2.812886	0
TC2N	0	0	0	-5.64989	0
WFDC1	4.388329	0	0	-2.87854	0
MYH10	-2.00516	-2.60405	0	0	0
GATA2	-3.15698	0	-3.83245	0	-3.96542
DGKA	0	0	3.552658	0	0
PDCD10	0	-2.12248	0	0	0
PRKCE	0	0	0	0	-3.73195
SH2B2	0	0	0	0	2.884399
TFF3	2.055269	3.312975	0	0	0
SERPINF2	-3.80776	0	-5.11472	-6.73975	-3.53702
CELSR1	0	4.245726	3.761182	0	0
SCARA5	-5.70063	0	-8.5801	-9.02178	-9.2662
TGFBR2	0	0	0	0	-6.21731
PRKACB	0	-2.37065	0	0	0
PTPN6	0	0	0	-5.15757	0
SOX15	0	4.992838	3.252009	-2.65174	0
CAV1	-4.1157	0	2.942347	3.055389	-4.33879
PDGFRB	0	0	0	0	-4.23707
HMOX1	-3.45552	2.418724	0	0	0

APOE	0	2.673705	0	0	8.638168
DCBLD2	0	0	5.575163	0	0
CCM2L	0	0	0	0	3.970996
JAK2	-1.90978	-3.07591	0	0	-4.71348
KRT6A	-3.38849	0	5.455358	-3.79927	-8.41348
WNT5B	0	0	2.848565	3.582366	0
F2RL3	0	0	-5.56458	-4.1391	0
PDGFD	0	0	-6.13257	0	-6.68398

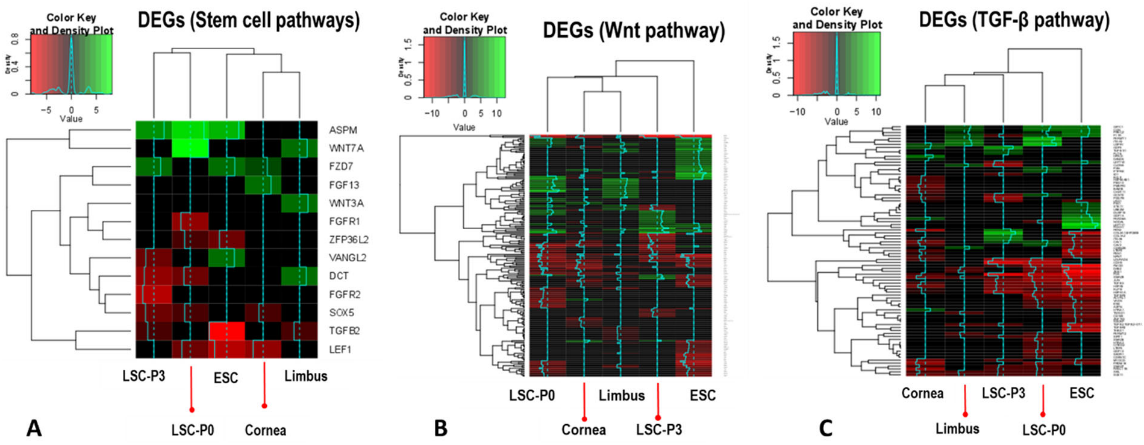

Supplementary Figure S1: DEGs of stem cell, TGF-β and Wnt signaling pathways. Heatmaps showing the differential expression of the genes belonging to stem cell pathway (A), Wnt signaling pathway (B), and TGFβ signaling pathway (C) expressed by cornea, limbus, LMSC-P3, LMSC-P0 and ESC (represented as columns) with respect to sclera. Each row corresponds to a single gene and the change in color from blue to red indicate enrichment of the genes specific to its particular pathway. Green (highlighted) boxes in the map represent the significant ($p<0.05$) up-regulation, red boxes represent significant down-regulation and black indicates insignificant ($p\geq0.05$) differences in gene expression, with respect to sclera (control).

Supplementary Table S6: DEGs of the Stem cell, TGF-β, and Wnt signaling pathways expressed by limbal stromal cells: table representing the log 2-fold change (relative to sclera) of each gene related to Stem cell, TGF-β and Wnt signaling pathways in the corneal and limbal tissues, limbal stem cells (LMSC-P0 and LMSC-P3) and embryonic stem cell, obtained from the RNA-Seq analysis.

Gene	LMSCP3	LMSCP0	ESC	Cornea	Limbus
**Stem cell signalling pathways**					
SOX5	-3.67946	-2.46716	0	-1.91442	0
FGFR2	-4.9523	0	0	0	0
WNT7A	-0.59201	8.163797	0	-1.5838	3.425595
DCT	-4.25981	-2.8202	0	0	3.018192
WNT3A	0	0	0	0	3.795823
VANGL2	-3.5835	0	3.47421	0	0
LEF1	0	-3.7229	-5.43219	-4.48318	0
TGFB2	-2.71935	0	-7.76926	0	-2.48183
ASPM	4.575232	6.133352	5.409098	0	0
FGF13	0	0	0	3.541949	0
FGFR1	0	-4.21262	0	0	0
ZFP36L2	0	-2.50251	-3.25638	0	0
FZD7	3.081614	0	3.632971	1.895351	0
**TGF-β Signalling pathway**					
PDGFB	-2.51477	0	0	-5.35556	0
FOS	-2.2883	0	-8.63988	-7.7411	-8.3862
ACVRL1	0	0	0	-3.94991	-5.0106
SOX11	-4.89062	-3.09812	-3.30244	-3.35014	0
HTRA3	0	0	0	-2.50128	0
FBN2	-5.52898	0	4.247491	0	0
FBN1	-1.98998	0	0	0	-5.63801
LTBP4	0	0	0	-2.60438	0
COL1A2	0	0	4.444916	0	-3.69186
ITGA8	-2.14716	0	4.00273	0	-5.15967
HSPA5	-4.20925	0	-4.14648	-4.38859	-5.84089
CD109	0	0	0	0	-3.8075
CITED2	0	0	0	-2.59367	0
GDF15	0	0	0	0	4.702094
SDCBP	0	0	0	0	-3.38985
HTRA1	2.904047	0	0	0	-3.65591
GIPC1	0	3.544737	0	4.62317	3.796651
PMEPA1	-4.11641	0	0	0	0
TGFBR3	0	0	0	-5.59763	-6.70427
CAV1	-4.1157	0	3.055389	2.942347	-4.33879

ITGA3	0	3.900411	0	5.326104	0
PEG10	-2.53551	0	0	0	0
FUT8	0	0	2.795841	0	0
PIN1	0	0	0	0	3.57466
GDF10	0	0	0	-3.71076	0
ID1	0	0	-2.49156	0	0
HSPA1A	-2.21032	0	-5.52505	-4.72885	-5.91505
TGFBR2	0	0	0	0	-6.21731
SKIL	-3.20579	-3.50819	-3.05667	-3.20496	0
COL3A1	0	0	6.867821	0	-7.03659
FERMT1	0	2.877748	0	7.000093	0
ZNF703	0	0	0	0	-3.35397
DAB2	0	-2.33844	0	-3.77695	-7.21337
THBS1	0	0	0	0	-7.81011
RASL11B	-5.26786	0	-3.15333	-2.89147	0
BAMBI	-3.24905	0	0	0	0
KLF10	-2.03719	0	-3.9004	-3.33433	-5.08257
LRG1	-1.90458	0	-4.72583	0	-2.86031
SMAD9	-5.16455	-2.33013	-3.02749	-5.68617	-7.41273
LDLRAD4	-2.75155	0	-6.02158	-6.55893	0
SMAD7	-3.37006	0	-2.62556	-3.26215	0
DUSP15	0	0	0	0	2.879924
CGN	0	2.918009	0	3.759838	4.579773
UBE2M	0	0	0	0	3.31988
NODAL	0	0	0	-2.73046	6.183362
JUN	0	0	-4.47018	-5.77923	-5.6497
CHST11	-3.53693	0	0	0	0
PARD6A	2.635301	0	0	0	5.863364
ENG	0	0	0	-2.4532	-4.46864
CAV2	-3.10544	0	0	2.393166	-4.05639
LEFTY2	0	0	-5.10998	0	3.527836
FMOD	0	0	-3.22283	-6.6357	-11.0862
TGFB2	0	-2.48183	-2.71935	0	-7.76926
HSP90AB1	-2.48869	0	0	0	0
CDKN1C	0	0	0	-3.38269	0
SKOR1	0	0	0	-3.12867	0
GDF5	2.890191	0	5.485205	0	0
F11R	0	3.108017	-2.42563	3.093681	3.847162
BCL9	-2.2746	0	0	0	0
CLDN5	0	2.228003	-5.76163	0	0
NPNT	-3.46855	0	0	0	-4.42115
STK11	0	0	0	0	3.496187

PRKCZ	0	2.140624	0	2.146655	4.449374
MYOCD	-4.71764	0	-3.57263	0	-3.35815
CDH5	0	0	-8.69042	-7.44243	0
GCNT2	-2.13899	0	-5.03088	2.990947	0
ASPN	0	0	0	-2.67927	-4.64704
VASN	0	0	0	-2.72378	-3.13072
SIRT1	0	-2.1351	-2.6792	-3.12729	0
PRDM16	-5.44768	0	0	-3.78611	-3.75311
TWSG1	0	-2.61025	0	0	-3.62704
DAND5	1.814331	0	0	0	0
USP9Y	3.356518	4.273204	0	7.896141	0
TGFB3	-2.5733	0	-3.74229	-4.44443	-8.29898
SMAD6	0	0	0	-4.33977	0
FERMT2	0	-2.58759	0	-2.76481	0
CDKN2B	0	2.907641	0	0	-4.56348
LEFTY1	0	0	0	0	9.322444
PXN	0	0	0	2.662403	0
HPGD	4.399931	0	0	0	0
ZEB1	0	-3.49661	0	-4.77607	-9.80105
FOXH1	0	0	0	0	7.789636
TGFB1I1	0	0	2.977089	0	0
LTBP2	-3.3408	0	0	0	-7.15267
**Wnt Signalling pathway**					
CDK14	0	0	-2.31221	0	0
TBX18	0	0	0	2.580988	-5.06074
FRAT2	0	0	2.31218	0	5.285946
GPRC5B	-2.9883	0	0	0	0
GRK5	0	0	0	0	-5.78719
SHISA2	0	0	0	-3.97169	0
FZD2	0	0	0	4.061838	4.180237
NDRG2	0	0	0	-5.68879	0
PLCB1	-3.09322	0	0	0	0
PPP1CA	2.312287	0	0	0	3.863012
MCC	0	0	0	0	-3.93398
SDC1	2.998155	0	3.01652	0	0
TRABD2B	0	-2.66551	-2.21209	0	-2.93616
LGR4	2.308584	0	0	0	0
NRARP	0	0	2.99954	-5.11657	0
DACT3	-3.55081	-1.93712	0	0	0
LGR6	0	2.320525	4.748744	0	0
PITX2	-4.33846	0	0	0	-7.34258

FGFR2	0	0	0	-4.9523	0
WNT7A	8.163797	-1.5838	3.425595	-0.59201	0
LRRK2	-4.06307	0	0	0	-5.95243
AP2S1	2.392438	0	0	2.646079	3.593135
RNF138	0	-2.2616	0	0	0
LEO1	0	0	-2.05488	0	0
SOX2	0	-2.3027	0	0	6.134517
DEPDC1B	5.924036	0	0	0	6.462453
WNT6	-3.03612	0	0	0	0
MDFI	3.018678	-3.33432	2.31346	-3.02404	0
SFRP4	-6.64555	-4.834	-4.27473	0	-8.39998
CSNK1G3	0	0	-2.31728	0	0
RSPO2	-5.80789	-5.13418	-2.3541	0	-4.87316
DKK1	3.620547	0	0	4.405673	0
FRAT1	0	0	2.19674	0	3.737593
GLI1	0	0	0	0	4.764286
ARNTL	-2.82946	0	0	0	-4.85424
IFT20	0	0	0	2.728297	0
RYR2	-2.32613	-2.52069	0	0	0
GPC3	-3.8297	-3.53904	0	-5.28435	3.365181
WNT9A	3.617588	2.833198	3.310776	0	0
CTNND2	-4.21157	-3.53517	-2.72186	0	0
DKK2	-4.25642	2.628251	0	0	0
FOXO1	-2.39216	0	0	-3.89163	0
NKD1	-2.68813	0	0	-6.53459	0
JUP	4.283844	0	4.222752	0	0
ARHGEF19	0	0	0	0	3.281424
WNT5A	0	2.665635	0	0	-4.82207
FZD9	0	0	3.19743	0	6.015469
CAV1	2.942347	-4.1157	0	3.055389	-4.33879
RNF43	3.266244	0	2.146427	-3.58755	0
PLCB3	2.322683	0	2.129307	0	3.599881
DACT1	-4.93764	-4.91435	-2.71743	-3.1841	-3.55244
HHEX	-5.64511	0	0	0	-2.88426
IGFBP6	0	3.921347	0	2.826069	-4.76709
ITGA3	5.326104	0	3.900411	0	0
DVL1	2.599764	0	0	0	3.819145
CCNE1	2.437851	-2.52508	0	0	4.155652
WNT7B	0	0	3.747699	0	0
WNT4	0	0	2.372188	-2.99886	0
WNT3A	0	0	3.795823	0	0
CSNK1G2	0	0	0	0	3.467835

RSPO3	-2.59502	-2.79537	0	-2.43678	0
PIN1	0	0	0	0	3.57466
PLCB2	0	0	0	-3.27618	0
WLS	0	-1.75371	0	0	-4.28858
WNT11	-6.37834	-4.97507	0	-5.67326	-5.08202
FRZB	-5.85794	-5.31014	0	-5.28809	-2.86674
SLC9A3R1	0	-1.8152	0	0	0
DACT2	0	0	4.065302	0	2.839232
FGF10	-4.22119	1.955867	0	0	0
FZD5	0	0	0	-4.95558	0
NXN	4.026208	0	3.249803	4.041176	4.125252
MAGI2	-4.57972	0	0	0	0
VANGL1	3.267428	0	0	0	0
VANGL2	0	0	0	-3.5835	3.47421
ZEB2	-4.26904	0	0	0	-9.2367
BICC1	0	0	0	0	-6.83572
JADE1	-2.87492	0	0	0	0
PFN1	0	0	0	0	3.689852
IGFBP4	-2.97632	0	0	0	0
CYLD	0	0	0	0	-5.29762
TCF7L2	-2.30213	0	0	0	0
TNN	-9.07401	-5.96824	-3.01638	-8.85936	-8.26415
GREM1	6.308248	-3.25508	2.10436	8.134189	0
COL1A1	0	0	0	3.86146	-3.83313
FERMT1	7.000093	0	2.877748	0	0
SOX4	0	-1.99354	0	0	0
ZNF703	0	0	0	0	-3.35397
DAB2	-3.77695	0	-2.33844	0	-7.21337
BMP2	0	0	0	-2.48081	-2.85949
BAMBI	0	-3.24905	0	0	0
PRKAA1	0	0	-2.24214	0	-3.42797
SFRP5	0	-3.33241	0	0	0
CPZ	-4.98025	0	-2.91251	0	0
TMEM198	0	0	0	0	3.611486
GNAO1	0	0	0	-3.3348	0
CCND1	4.090886	0	0	3.270751	0
APCDD1	0	0	0	0	-3.67854
SOX7	0	-2.03853	0	-6.47092	-4.96516
TIAM1	0	0	2.624656	0	0
FUZ	0	0	0	0	4.084022
APOE	0	0	2.673705	0	8.638168
DLX5	-3.88005	-2.47262	0	0	-3.44238

TMEM88	-6.13849	0	0	-6.48777	0
PPP3CA	0	0	0	0	-3.16652
NKD2	-4.00004	0	0	-3.48492	0
RNF146	0	0	-2.36491	0	0
MBD2	0	0	0	0	-3.36297
TNIK	0	-2.75701	0	0	0
RBPJ	0	0	-2.17311	0	0
GNG2	-2.65049	0	0	0	0
LEF1	-3.7229	-4.48318	0	0	-5.43219
FOXO3	-2.48049	0	0	0	-4.19983
NOG	0	0	0	-2.37342	0
PSMD2	2.695562	0	0	0	0
WNT2	0	0	0	7.8247	0
WWTR1	0	-2.1292	0	0	-5.29775
MARK1	0	-3.30635	0	-4.86852	0
PTPRO	-3.82579	0	0	0	0
WNT2B	-2.3622	0	0	0	-4.5358
PARD6A	0	2.635301	0	0	5.863364
CELSR2	4.09746	0	3.701377	0	0
PSMD12	0	0	-2.36553	0	0
SULF1	0	-2.23799	-2.3108	4.836812	-3.84404
TRPM4	2.191288	1.804944	0	0	0
CTNNBIP1	3.175764	0	2.489421	0	4.37156
KLF4	-4.05748	0	0	-5.55662	-6.85836
SOX17	0	-6.15503	0	0	0
HBP1	0	0	0	0	-3.92352
FZD6	0	-2.15041	0	0	0
PDE6G	0	0	0	0	3.333578
SFRP1	0	0	0	-3.94856	0
FGF8	0	0	0	0	4.648729
PRICKLE2	0	0	0	0	-3.3683
PIAS4	0	0	0	0	3.457345
MYOC	-11.7551	-3.66614	-2.60433	-12.2447	-12.6236
DIXDC1	-2.9729	0	-2.60599	0	-4.76062
ASPM	6.133352	0	0	4.575232	5.409098
BCL9	0	-2.2746	0	0	0
STK11	0	0	0	0	3.496187
GRK6	2.247842	0	2.55516	0	4.630793
CPE	-3.74187	-4.56895	0	0	-3.4578
CTHRC1	0	-2.43876	0	3.733465	0
NR4A2	-5.08336	-4.66994	0	-9.26053	-9.42924
GRHL3	3.335591	0	3.762522	-4.47326	0

EGR1	-7.11579	-2.94803	0	-4.85668	-9.15295
EGF	2.570847	0	3.032097	0	0
PPP2R1A	0	0	0	0	3.681628
CDC73	0	0	-2.19064	0	0
FZD1	-2.45931	0	0	0	-4.30458
PSMB10	3.273024	0	2.432979	3.168493	3.073423
DDIT3	-5.10798	-2.3419	0	-2.87561	-3.38352
FOXL1	0	-2.45934	0	0	-3.7801
KREMEN1	0	0	0	0	-3.62708
NFKB1	0	-1.97001	0	0	-4.82035
LRP1	-3.32579	0	0	0	-4.14957
CD24	5.714639	-3.29942	0	-3.05475	5.68427
APC	0	0	-2.28793	0	0
RSPO1	-4.02554	0	0	0	-4.0698
RARG	0	0	2.532582	0	0
DVL2	0	0	0	0	3.282172
NLE1	2.256862	0	0	0	5.186813
TNRC6C	-3.69215	0	0	0	0
WNT5B	2.848565	0	0	3.582366	0
WIF1	-4.58262	-3.17714	0	0	0
TLE3	0	0	0	-2.86358	0
TLE2	-3.90063	0	0	-2.85914	0
STK3	0	0	-2.19043	0	0
PSME4	0	-2.09792	0	0	-3.46479
NFATC4	-2.76936	0	0	0	0
NFATC1	-4.56311	0	0	-3.15884	0
WNT10A	3.673524	0	4.450882	0	0
SOX10	-3.27508	-3.47425	0	-3.45931	0
MDFIC	0	0	-2.24866	0	-5.82661
CCDC88C	0	-1.83703	0	-5.81313	0
CELSR1	3.761182	0	4.245726	0	0
VPS26A	0	0	-2.07267	0	0
P2RX5	2.721445	0	0	3.014619	0
GPC6	-2.91287	-4.20522	-3.12343	0	0
TSPAN12	0	-2.60829	0	-3.71885	0
AXIN2	-5.76546	0	0	0	0
FERMT2	-2.76481	0	-2.58759	0	0
KREMEN2	3.209791	0	0	0	5.056132
SFRP2	-7.74245	-5.4964	-2.59633	-6.31747	0
CDH2	0	0	0	3.043564	0
CDH3	6.615209	0	4.704429	0	6.076329
DRAXIN	0	0	0	0	6.992601

PSMC6	0	0	-2.09296	0	0
FZD4	-3.2931	0	0	0	-3.0429
FZD10	-4.36757	0	0	0	0
PDE6B	0	2.501113	0	0	4.211281
PRKAA2	0	0	0	-3.07973	0
TLR2	0	0	0	0	-5.67379
ROR1	0	-2.23853	0	2.76985	0
PSMB5	2.945327	0	0	2.438757	3.393788
SMO	0	-2.3963	0	0	3.674392
DRD2	0	3.442471	0	0	0
WNT16	-4.60017	-2.18488	0	-4.00975	-5.09831
DKK4	3.34473	0	4.119286	0	0
HIC1	0	0	0	0	-5.74552
SNAI2	0	0	0	0	-6.70694
MLLT3	0	-1.989	-2.18898	0	0
GPC4	-3.54738	0	0	-4.23619	0
TGFB1I1	0	0	0	2.977089	0
PYGO1	-3.25998	0	-2.59361	0	-5.17901
MAD2L2	0	0	0	0	5.320885
CMAHP	-4.6791	0	0	0	0
LRP4	0	2.399472	0	0	0
FZD7	0	1.895351	0	3.081614	3.632971
RSPO4	0	-4.91874	0	0	-3.91532
